# Baseline imaging characteristics and early structural changes in macula on rhegmatogenous retinal detachment

**DOI:** 10.1038/s41598-024-51183-8

**Published:** 2024-01-16

**Authors:** Alberto Quarta, Matteo Gironi, Maria Ludovica Ruggeri, Agbeanda Aharrh-Gnama, Annamaria Porreca, Rossella D’Aloisio, Lisa Toto, Marta Di Nicola, Rodolfo Mastropasqua

**Affiliations:** 1 Department of Sciences, Ophthalmology Clinic, National Center of High Technology in Ophthalmology, Gabriele D’Annunzio University, Via dei Vestini, 66100 Chieti, Italy; 2 Department of Medical Oral Science and Biotechnology, G. d’Annunzio University of Chieti-Pescara Chieti, Via dei Vestini 31, Chieti, Italy

**Keywords:** Medical research, Biomarkers

## Abstract

Animal models have demonstrated that structural changes affect the macula during peripheral rhegmatogenous retinal detachment. This study aimed to assess photoreceptors, retinal and choriocapillaris perfusion in non-macula involving rhegmatogenous retinal detachment by analyzing en-face images from structural OCTA segmented at the ellipsoid zone (EZ) level, calculating (1) “normalized” reflectivity as a surrogate biomarker of photoreceptor damage (2) perfusion density (PD), vessel length density (VLD) and vessel diameter index (VDI) of superficial capillary plexus (SCP) and deep capillary plexus (DCP) (3) perfusion density of choriocapillaris (PDCC). Twenty-one eyes affected by macula-on rhegmatogenous retinal detachment (RRD) were enrolled at the University “G. d’Annunzio”, Chieti-Pescara. The fellow unaffected eye was used as control. The mean age at the onset of RRD was 60.09 ± 10.22 (range 34–83). Compared with fellow eyes, we found lower EZ “normalized” reflectivity in macula-on (0.42 ± 0.15 in fellow eyes and 0.31 ± 0.09 in macula on p = 0.004). The affected eye was also characterized by impaired perfusion in SCP (17.26 ± 3.34% in macula on and 20.56 ± 3.62% in the fellow eye p = 0.004) and CC (50.21 ± 6.20% in macula on the eye and 57.43 ± 6.20% in the fellow eye p = 0.004). Macula-on rhegmatogenous retinal detachment has subclinical changes in photoreceptors, SCP, and CC. Future longitudinal studies should evaluate if early changes could impact post-operative macular function.

## Introduction

Rhegmatogenous retinal detachment (RRD) is a potentially blinding condition^1^. The mechanical detachment from the retinal pigmented epithelium (RPE) starts a chain reaction of cellular changes, reflecting anatomical abnormalities in retinal architecture well described in animal studies but not humans^2–5^.

Generally, RRD is divided into ''macula-on'' and ''macula-off'' according to the attached or detached macula. Although macula-off RRD often underlines worse visual acuity at presentation and visual prognosis, macula-on could hide structural non-apparent changes that could justify initial visual impairment and difficulties in reaching an optimal functional recovery^6–9^.

During retinal detachment, early metabolic and vascular changes can occur in the inner and outer retina^10–12^. Retinal vascular plexuses nourish the inner retinal layers, and choroid and choriocapillaris (CC) do the same for the outer retinal layers. Nonetheless, some authors demonstrated that capillary plexuses may contribute to the photoreceptor metabolic demand^13^.

Optical coherence tomography (OCT) and optical coherence tomography angiography (OCTA) are part of the multimodal imaging tools that help us understand the near-in vivo microscopic changes of retinal architecture^14^.

En-face OCT enables qualitative and quantitative topographical measurements at several depths.

The ellipsoid zone (EZ) corresponds to the inner segment/outer segment of the photoreceptor layers, and en-face images can spot irregularities of EZ, seen as hyporeflective areas. Previous works demonstrated how EZ reflectivity may indicate photoreceptor integrity, representing a possible prognostic visual biomarker in different conditions^15,16^.

This study aimed to assess imaging characteristics of the macula in retinal detachment at presentation, evaluating near in-vivo cellular subclinical changes of photoreceptor status through EZ normalized reflectivity. Secondly, vascular qualitative and quantitative changes of retinal capillary plexuses and choriocapillaris were evaluated.

## Methods

### Study participants

This prospective case–control study included 21 consecutive patients (21 eyes) with macula-on RRD (study group) enrolled at University “G. d’Annunzio”, Chieti-Pescara, Italy, between February 2023 and March 2023. The fellow healthy eye was used as the control group. This study was approved by the institutional review board and adhered to tenets of the Declaration of Helsinki. Informed consent was obtained from all patients before surgery.

All patients underwent complete ophthalmologic examination before surgery. Examinations included assessment of best corrected visual acuity (BCVA), intraocular pressure (IOP) with Goldman applanation tonometry, fundus examination with indirect ophthalmoscopy after 1% tropicamide instillation, and multimodal imaging with OCT and OCTA on the day of surgery.

Inclusion criteria were: (1) primary macula on RRD, according to Klaas et al.^17^, with a distance macula-detachment > 6 mm^17^, (2) BCVA > 0.1 logMAR, (3) symptoms at presentation that lasted less than a week including temporal photopsias, sudden or progressive perception of floaters and peripheral scotoma.

Exclusion criteria were: (1) prior intraocular surgery except for uncomplicated phacoemulsification, (2) other concomitant retinal diseases including diabetic retinopathy, high myopia (AxL > 26 mm), age-related macular degeneration, inherited retinal dystrophies, retinal vascular diseases and vitreomacular disorders like epiretinal membrane and macular hole (3) glaucoma and other optic neuropathies, (4) complex RRD defined as the presence of proliferative vitreoretinopathy (5) non-rhegmatogenous retinal detachment in the contest of diabetic retinopathy, uveitis, ocular tumors or exudative.

### Imaging

All eyes underwent multimodal imaging examination with the same spectral domain OCT (SD-OCT) Spectralis HRA + OCT imaging device, with OCT Angiography Module (Heidelberg Engineering, Heidelberg, Germany) taking a 30° radial scan centered on the fovea. Additional OCTA software imaging was done using SS-OCTA Plex Elite 9000 (Carl Zeiss Meditech) with a 3 × 3 mm scan area centered on the fovea.

OCTA provides 3-dimensional maps of the SCP, DCP, and CC, exported separately as en-face images to be analyzed.

### Image analysis

Preoperative SD-OCT and SS-OCTA scan morphological data were evaluated and reviewed independently by independent researchers (AQ, MG). If no intergrader agreement could be obtained, the final agreement was confirmed by consulting a senior investigator (RM). Only images with sufficient signal strength index (SSI) were included (> 8/10).

**Figure 1:**
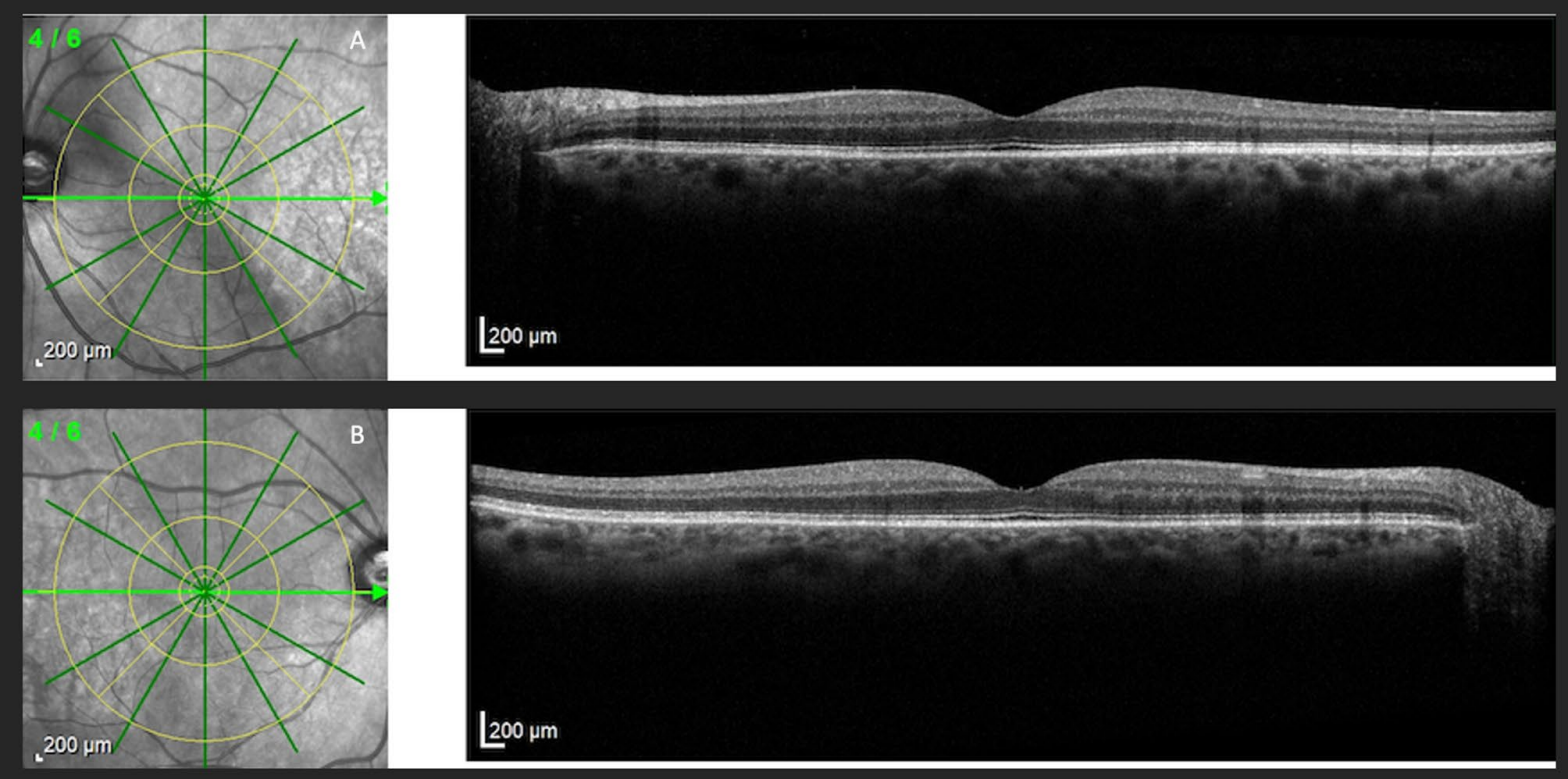
Near-infrared reflectance images show the location and direction of the structural B-scan, with the superimposed ETDRS grid to assess the macula-on the state (**A**). We can notice the regular appearance of the macula and choroid in both the RRD eye (**A**) and fellow eye (**B**).

The ETDRS grid historically used in the ETDRS study available on Heyex 2 Software was used to assess the level of macular involvement on SD-OCT scans. The ETDRS overlay was correctly focused on the fovea using the Infrared (IR) and B-Scan images as morphological correlates (Fig. 1).

The placement of the foveal area in B-Scan images and the topographical relationship to the retinal vasculature and optic nerve head in IR images were employed as typical microstructural and anatomical markers to pinpoint the fovea.

The en-face images were processed following an algorithm previously described^18^. Briefly, a circular region of interest centered on the fovea with a radius of 1.5 mm was selected for measurements. The radius dimension was justified because of the limited lateral resolution of the face images.

Investigating EZ “normalized” reflectivity, the EZ en-face image was exported and then imported into ImageJ software version 1.50 (National Institutes of Health, Bethesda, MD; available at http://rsb.info.nih.gov/ij/index.html).

The mean brightness of the EZ en-face image was measured. A well-known image processing algorithm was used to normalize the signal among subjects to eliminate various disturbance factors^19–21^. Vitreous and retinal nerve fiber layers are used as references for the dark and bright structures to be compared with EZ reflectivity. Since previously excellent interobserver agreement for the EZ ''normalized'' reflectivity algorithm has been demonstrated, a formula was applied to determine EZ ''reflectivity'' after testing the mean brightness of each structure for each eye^18^.

En-face OCTA images segmented at the SCP, DCP, and CC levels were imported into ImageJ software to calculate the perfusion density of the capillary plexuses. After the binarization of each slab exported with the previous thresholding algorithm, perfusion density (PD) was calculated, while ''Phansalkar'' threshold was used to calculate CC perfusion density (PDCC)^22^.

Vessel length density (VLD) was measured after skeletonization on binarized images of SCP and DCP. Vessel diameter index (VDI), a surrogate measure of average vessel caliber, was calculated by dividing the area in the binarized image by that in the skeletonized image.

### Statistical analysis

Using both eyes for each patient created nested paired data (due to the within-subject correlation). The power calculation was thus based on a two-sided paired *t*-test testing the null hypothesis that there would be no difference (on average) in within-subject mean EZ “normalised” reflectivity changes between the paired eyes. Twenty patients were required to detect an effect size of 0.7 with 80% power at a 5% significance level. The Shapiro–Wilk test was performed for all variables to detect departures from a normal distribution. Means and standard deviation (SD) were used as descriptive statistics for all quantitative variables. Data were analyzed using a paired two-tailed Student’s t-test. All statistical tests were 2-sided with a significance level set at p ≤ 0.05. All statistical analyses were performed using Statistical Package for Social Sciences (version 22.0; SPSS Inc, Chicago, Illinois, USA).

### Ethics approval

The Institutional Review Board Committee of Gabriele D’Annunzio University of Chieti-Pescara at SS. Annunziata Hospital (OCTARD-1-2023).

### Informed consent

Patient consent was obtained.

## Results

### Patient characteristics

Twenty-one patients (21 eyes) with RRD and their fellow eyes were analyzed. The mean ± SD age was 60.09 ± 10.22 (range 34–83). All patients had a BCVA > 0.1 logMAR. Among patients, 6 (28%) were pseudophakic. The mean IOP was 17.00 ± 1.00 mmHg (range 15–19). The mean IOP in fellow eyes was 16.67 ± 1.46 mmHg (range 14–19). No statistical difference was reported between the two (p = 0.273). En-face optical coherence tomography and optical coherence tomography angiography.

**Figure 2:**
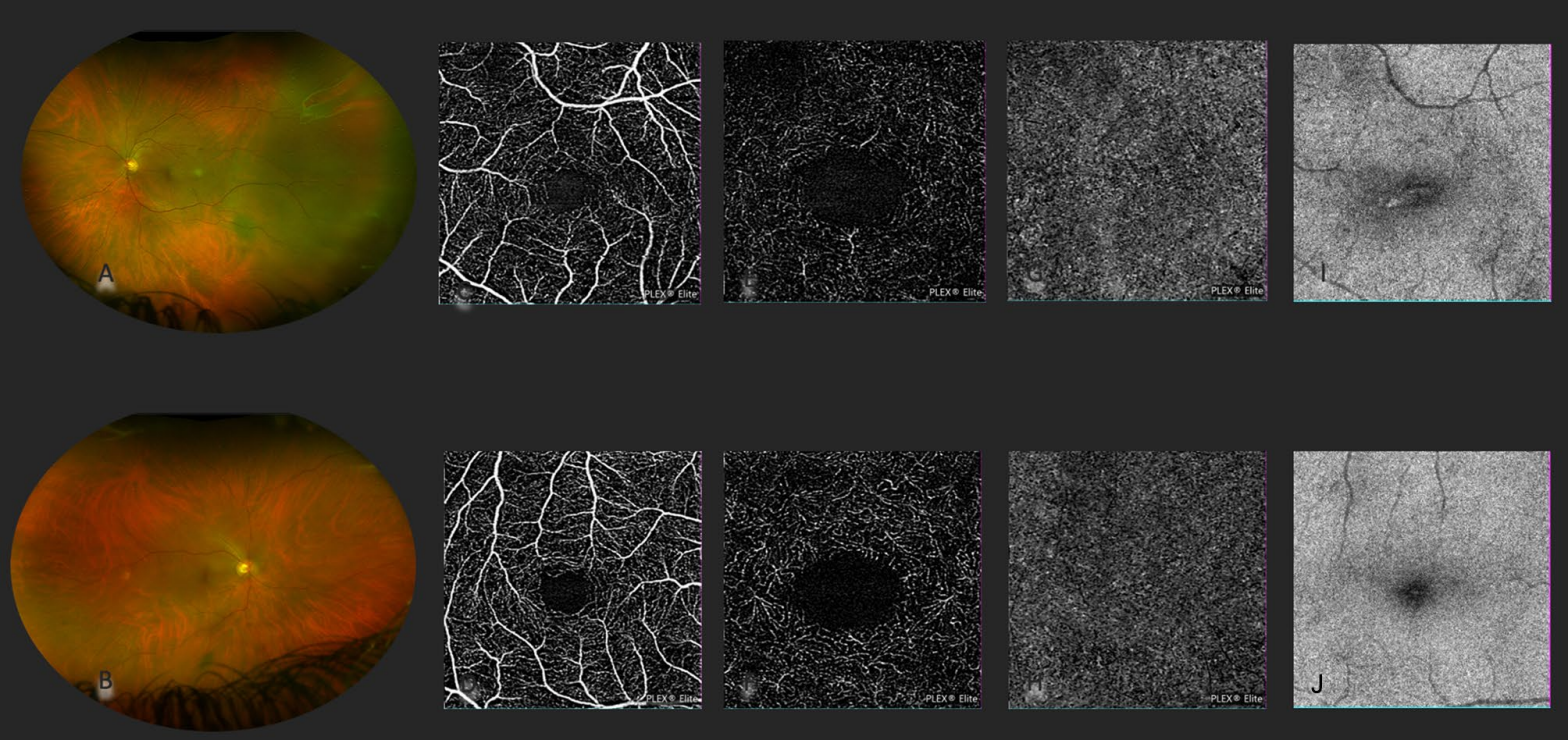
Multimodal imaging of a macula on RRD and fellow eye. Fundus pseudocolor image (**A**) shows temporal non-macula involving rhegmatogenous retinal detachment compared to the fellow eye (**B**). En-face image analysis from OCTA shows perfusion changes in comparison with a fellow eye for superficial capillary plexus (**C**,**D**), deep capillary plexus (**E**,**F**), and choriocapillaris (**G**,**H**). EZ reflectivity (**I**) also shows substantial changes in the macula-on eye compared to its fellow (**J**), highlighting subclinical photoreceptor damage.

We divided image analysis into two groups: OCTA analysis, where capillary vascular plexus data were reported, and en-face OCT analysis, where PDCC and EZ ''normalized'' reflectivity were reported (Fig. 2).

In SCP evaluation, the mean PD was 17.26 ± 3.34% and 20.56 ± 3.62% in the macula-on and fellow eye, respectively (p = 0.004). The SCP VLD was 2.69 ± 0.59% in the macula and 3.36 ± 0.70% in the fellow eye group (p = 0.002). The SCP VDI was 6.45 ± 0.36% in the study group and 6.16 ± 0.32% in the control group (p = 0.010).

For the DCP PD and VLD mean ± SD, lower values were found in the macula-on eyes (15.69 ± 3.34% vs. 17.12 ± 3.97% with p = 0.213 and 2.82 ± 0.80% vs 2.93 ± 0.75% with p = 0.643) DCP VDI values were higher in fellow eyes (5.88 ± 0.33% vs 5.67 ± 0.62% p = 0.185).

The mean for PDCC was 50.21 ± 6.20% in the study group and 57.43 ± 6.20% in the control group (p = 0.004). EZ ''normalized'' reflectivity mean value was 0.31 ± 0.09 in the macula on eyes and 0.42 ± 0.15 in the fellow eye group (p = 0.004) (Table 1).

**Table 1:** Tested variables in macula on and fellow eye.

	Macula on eye	Fellow eye	P value
SCP perfusion density (%)	17.26 ± 3.34	20.56 ± 3.62	0.004
SCP vessel length density (%)	2.69 ± 0.59	3.36 ± 0.70	0.002
SCP vessel diameter index (%)	6.45 ± 0.36	6.16 ± 0.32	0.010
DCP perfusion density (%)	15.69 ± 3.34	17.12 ± 3.97	0.213
DCP vessel length index (%)	2.82 ± 0.80	2.93 ± 0.75	0.643
DCP vessel diameter index (%)	5.67 ± 0.62	5.88 ± 0.33	0.185
CC perfusion density (%)	50.21 ± 6.20	57.43 ± 6.20	0.004
EZ ‘’normalized’’ reflectivity	0.31 ± 0.09	0.42 ± 0.15	0.004

*SCP* superficial capillary plexus, *DCP* deep capillary plexus, *CC* choriocapillaris, *EZ* ellipsoid zone.

## Discussion

Rhegmatogenous retinal detachment is a condition that needs prompt surgical intervention. Although the macula-on state gives a good visual prognosis, some early subclinical changes could occur since visual deficits are not restricted to the detached retina but are also present in the non-detached tissue^6–9^. Following the cited algorithm, this is the first study demonstrating a near-in vivo photoreceptor alteration accompanied by perfusion changes in the macula-on RRD.

Previous clinical studies showed that some vascular alterations exist in the setting of peripheral detachment^10,23–27^. Exploring structural changes not visible with conventional B-scan images, our study assessed the baseline condition, evaluating the pathophysiological changes of the attached macula during the peripheral detachment. Macula-on eyes were characterized by a reduction in both PD and VLD. While VLD is based on an image with a vessel of 1 pixel width in which the final measurement is equally influenced by thicker and thinner vessels, PD measures the total area of vessels. The two values permits calculating the VDI, a surrogate biomarker of average vessel caliber. Since we found a higher value in VDI, we speculate that early hypoperfusion and vascular damage may be manifested with increased average vascular diameter. In this setting, we found that CPD was reduced, which could be relevant since it might increase the expression of vascular endothelial growth factor from RPE^28^.

Four potential hypotheses may explain the involvement of retinal capillary plexuses, choriocapillaris, and photoreceptor alteration in attached macula during peripheral retinal detachment: (1) increased peripheral vascular resistance from blocked retinal circulation in detached areas, (2) Muller cells hyperactivation by producing a pool of cytokines, (3) the role of subretinal fluid in the periphery made by many factors and among them, endothelin-1 represents a powerful vasoconstrictor, and (4) altered oxygen supply from the choriocapillaris because of the subretinal fluid as steric hindrance. The macula detachment could be only the final step of the above-mentioned mechanisms.

Sato et al. reported the mean circulation time in detached and undetached areas, demonstrating a slower blood flow in detached ones^29^. Using ultrawide-field imaging and OCTA, Feng described qualitative and quantitative changes in retinal vasculature in both RRD and non-RRD areas^30^.

Our work demonstrates that a SCP impairment is objective. We add information about the microvascular quality of SCP by reporting that VLD and VDI are also affected in these patients.

Conversely to previous studies^24,31^, our results show a non-significant reduction of PD DCP in the macula-on eyes. This might be due to the very initial state of the disease. The progression of the detachment might influence DCP values since Savastano et al. have suggested the presence of vertical anastomoses between the DCP and SCP. The initial compensatory response from SCP with autoregulation may protect the DCP in the early phase of RRD^32^.

Another reason for different results for DCP relies on indexes considered during analysis. In theory, vessel length density can provide superior precision because it is unaffected by size differences between the OCT beam focal spot and OCTA pixel widths compared to capillary diameters. This measure requires accurate skeletonization of the capillary network and near-seamless vessel continuity, a high sampling density (pixel width lower than the focal point diameter) and great image quality.

This study demonstrates choriocapillaris perfusion impairment and an initial injury to photoreceptors in the macula-on RRD. Many factors may be involved in this phenomenon.

Direct or indirect detachment-induced hypoxia could trigger Muller cell proliferation and hypertrophy^33,34^. Soluble factors may influence Muller cell gliosis and microvascular perfusion; in particular, those released by the detached tissue and diffused into the surrounding areas are likely to be implicated^35^.

Subretinal fluid in the peripheral detached retina could also play a role in hypoperfusion and subclinical retinal degeneration. Among released cytokines, endothelin-1 may be essential in perfusion impairment in the peripheral retina and the surrounding areas^36,37^. Moreover, subretinal fluid could prevent regional oxygen diffusion from the periphery, which could spread among the non-detached area, leading to subclinical photoreceptor damage. Performing analysis on a region of interest, ''Normalized'' reflectivity images solve restricted lateral resolution and intrasubject factors that affect structural brightness. We demonstrate that quantitatively, lower EZ reflectivity affects the macula-on eyes.

Our study has some limitations. The sample size is relatively small. We do not have additional angiographic data to add dynamic vascular information. In addition, the subretinal fluid composition was not analyzed, which would help cytokine analysis. Further randomized longitudinal studies are needed to assess the role of these pathological changes in qualitative and quantitative vision recovery after surgery. Additional studies should focus on the impact of surgical timing on these early changes.

This algorithm is well-described in patients with vitreous body because they take it as a reference, so it could be challenging to apply in the eyes after vitrectomy. Our study spotlighted macular photoreceptor changes during peripheral detachment, so further methods should be developed to understand how photoreceptors could change after surgery.

In conclusion, our study assessed the presence of SCP and CC impairment with a subclinical photoreceptor alteration by demonstrating lower ''normalized'' reflectivity of EZ. Future analysis could clarify if this data might influence the history of the macula-on retinal detachment. However, this could be one of the reasons for post-operative suboptimal BCVA and contrast sensitivity. Further investigations should clarify if photoreceptor degradation and choriocapillaris hypoperfusion could play a role in restoring visual acuity after surgical procedures.

## Data Availability

The datasets used and analyzed during the current study are available from the corresponding author upon reasonable request.
